# Weight change is significantly associated with risk of thyroid cancer: A nationwide population-based cohort study

**DOI:** 10.1038/s41598-018-38203-0

**Published:** 2019-02-07

**Authors:** Hyemi Kwon, Kyung-Do Han, Cheol-Young Park

**Affiliations:** 1 Division of Endocrinology and Metabolism, Department of Internal Medicine, Kangbuk Samsung Hospital, Sungkyunkwan University School of Medicine, Seoul, Republic of Korea; 2 Department of Biostatics, Catholic University College of Medicine, Seoul, Republic of Korea

## Abstract

Obesity is a risk factor for many cancers including breast, esophageal, colon, and thyroid cancer. We aimed to evaluate the association of thyroid cancer with body mass index (BMI), waist circumference (WC), and weight change. This nationwide population-based cohort study included 11,323,006 adults who joined the national health screening program. Weight change was defined as the difference between the weight of the subjects measured during the study period and the weight at the time of four years ago. For evaluating the association between the weight change and the risk of thyroid cancer, subjects without weight change for four years were defined as the reference group. Mean age was 50.1 ± 13.7 years and 44% were female. In total, 50,464 subjects (0.4%) had newly-diagnosed thyroid cancer. After multivariable analyses, the incidence of thyroid cancer increased significantly in subjects with larger WC as well as higher BMI (P < 0.001 and P < 0.001, respectively). In subjects who were lean and became obese, the incidence of thyroid cancers increased significantly (hazard ratio [HR] 1.15 [1.11–1.19]). In subjects who were obese and became lean, the incidence of thyroid cancers decreased significantly (HR 0.89 [0.86–0.93]). These results demonstrated that higher BMI and larger WC were significantly associated with an increased risk of thyroid cancer. Weight gain in lean subjects was associated with an increased risk of thyroid cancer, and weight reduction in subjects with obesity was associated with a decreased risk of thyroid cancer.

## Introduction

Obesity is a medical condition defined as excessive accumulation of body fat^1,2^. It is a well-known risk factor for several cancers including breast cancer, endometrial cancer, colon cancer, esophageal adenocarcinoma, prostate cancer, liver cell carcinoma, leukemia, non-Hodgkin lymphoma, and melanoma^3–6^. Obesity has become more prevalent worldwide and is a major public health issue^7^. In Korea, the prevalence of obesity has steadily increased from 28% in 2006 to 32% in 2015 and the prevalence of abdominal obesity has also steadily increased from 18% in 2009 to 21% in 2015^8^. In the Unites States, the prevalence of obesity increased from 13% to 35% between 1960–1962 and 2011–2012^9,10^. Similarly, the incidence of thyroid cancer has been increasing throughout the world during the last decades^11–13^.

Recently, several studies have reported that obesity is positively associated with an increase in the risk of thyroid cancer^3,14–21^. Because body mass index (BMI) is a widely used measure for obesity, the association between higher BMI and the risk of thyroid cancer is well known^3,14–21^. Other anthropometric markers such as waist circumference (WC) and weight gain could reflect central adiposity and body fat mass^21–25^. However, the association between WC and the risk of thyroid cancer is conflicting^18,21,26,27^. A study from the United States demonstrated that subjects with large WC had significantly higher incidences of thyroid cancer in both males and females^26^. However, another study demonstrated that there was no significant association between WC and risk of thyroid cancer^27^. Weight gain is dynamic and represents the accumulation of body fat mass rather than lean mass^25,28^. It was related to unfavorable fat distribution, reduced metabolic efficiency, and the increased risk of adiposity-related cancers such as breast cancer, endometrial cancer, ovarian cancer, colon cancer, and kidney cancer^25^. However, the association between weight change and risk of thyroid cancer remains debatable^26,27,29–31^. Previous studies had suggested a positive association between weight gain and risk of thyroid cancer^26,29^. while other studies had demonstrated no significant associations^27,30,31^.

In the present study, we aimed to evaluate the associations of thyroid cancer with BMI, WC and weight change using a large population-based cohort from on the Korean National Health Screening database.

## Methods

### Study design and subjects

In this nationwide population-based cohort study, 11,501,967 subjects age 20 years or older who joined the national health screening program from 2009 to 2012 were included. This retrospective cohort data was collected by the Korean National Health Insurance Service (NHIS), which is managed by the government^32–34^. Most of Korea’s 50 million in population is included in the Korean NHIS, because the Korean government provides a health insurance system, one of a social security system^32–34^. The national health screening program enables all insured Koreans who are at least 40 years old, along with their dependents to have a universal health checkup every 2 years^33–35^. Retrospective cohort data using the national health screening database, which represent most of the Korean population, includes medical information such as health behaviors using self-reporting questionnaires, bio-clinical variables from anthropometric measurements and laboratory tests for blood and urine, medical history, medical treatment, and information on insurance claims from reported International Classification of Diseases, Tenth Revision (ICD-10) codes^32,34^. This study was approved by the Institutional Review Board of Kangbuk Samsung Hospital. All research was performed in accordance with relevant guidelines and regulations. Informed consent requirement was waived because personal identifying information was not accessed.

### Anthropometric measurements

Measurements of height, weight, WC, blood pressure and laboratory data were collected. BMI was calculated using the following formula; the weight in kilograms divided by the square of the height in meters (kg/m^2^). We divided BMI into 5 categories based on the guideline of the Korean Society for the Study of Obesity (KSSO) and recommendations of World Health Organization (WHO) of Asia-Pacific perspective^2,36^: underweight (<18.5 kg/cm^2^), normal-weight (18.5–22.9 kg/cm^2^), overweight (23.0–24.9 kg/cm^2^), obesity (25.0–29.9 kg/cm^2^), and severely obese (≥30.0 kg/cm^2^)^2,36^. WC, measured at the superior border of the iliac crest, was categorized into 6 levels as follows: WC for males, <80, 80–85, 85–90, 90–95, 95–100, ≥100 cm, WC for female, <75, 75–80, 80–85, 85–90, 90–95, ≥95 cm. Weight change was defined as the difference between the weight of the subjects measured during the study period and the weight at the time of four years ago^37^. Because most of the insured Koreans have a universal health checkup every 2 years^33–35^, it was available that information on the body weight at the time of four years ago in subjects who received a health checkup between 2009 and 2012^37^.

Basic information and health-related behaviors like smoking (non-smoker, ex-smoker, or current smoker), alcohol intake (none, 1–3 times/month, ≥1 time/week), and regular physical activity were collected with standardized self-reporting questionnaires^32^.

### Definition of disease condition

Patients with thyroid cancer were defined as having the ICD-10 code C73, and we excluded patients who were diagnosed with thyroid cancer prior to the study period. Diabetes was defined as a fasting blood glucose level ≥126 mg/dL (≥7 mmol/L), or the presence of one or more claims per year for anti-hyperglycemic medications with ICD-10 codes E10–14^33^. Hypertension was defined as systolic blood pressure (SBP) ≥ 140 mmHg or diastolic blood pressure (DBP) ≥ 90 mmHg, or the presence of one or more claims per year for anti-hypertensive medications with ICD-10 codes I10-I15. Dyslipidemia was defined as total cholesterol levels ≥240 mg/dL (≥6.22 mmol/L), low-density lipoprotein (LDL)-cholesterol ≥ 100 mg/dL (≥2.59 mmol/L), triglycerides ≥ 150 mg/dL (≥3.88 mmol/L), high-density lipoprotein (HDL)-cholesterol < 40 mg/dL (<1.04 mmol/L) in men or <50 mg/dL (<1.29 mmol/L) in women, or the presence of one or more claims per year for anti-dyslipidemic medications with ICD-10 code E78. Regular physical activity was defined as more than one day of moderate-intensity or vigorous intensity in a week using self-reporting questionnaires. Hypothyroidism and hyperthyroidism were defined using ICD-10 codes (E02, E03, E05, and E06.3), medication, and treatment, as our previous study^38^.

### Statistical Analysis

For statistical analysis, SAS version 9.3 (SAS Institute Inc., Cary, NC, USA) was used. We excluded subjects who were diagnosed as thyroid cancer at the first years of follow-up and censor date was 31th December 2015. Considering the effect of hypothyroidism or hyperthyroidism on weight, we excluded participants taking thyroid hormone because of hypothyroidism or undergoing treatment because of hyperthyroidism at the time of enrollment of this study. Continuous variables are expressed as means ± standard deviations. Categorical variables are presented as numbers and percentages. The incidence rate of thyroid cancer was calculated by dividing the number of incident cases by the total follow-up period and presented per 1,000 person-years. The risk of thyroid cancer was analyzed with a Cox proportional hazard model in order to evaluate hazard ratios (HRs) with 95% confidence intervals (CIs) according to BMI, WC, and weight change. Multivariable analyses were adjusted for age, sex, smoking status, alcohol intake, regular physical activity, diabetes, hypertension, and dyslipidemia. For evaluating the association between the weight change and the risk of thyroid cancer, subjects without weight change for four years were defined as the reference group. *P* for trend analyses according to BMI and WC were performed with a general linear model. All *P-*values were two-sided and *P* < 0.05 was considered statistically significant.

## Results

### Baseline characteristics of subjects

In total, 11,323,006 adults were included in this study. Baseline characteristics of subjects are shown in Table 1. The mean age was 50.1 ± 13.7 years and 44% of the subjects were female. The mean height was 163.6 ± 9.3 cm and the mean weight was 64.1 ± 11.5 kg. The mean BMI was 23.8 ± 3.2 kg/m^2^. In total, 3.3% of subjects were underweight (BMI < 18.5 kg/m^2^), 37.8% were normal-weight (BMI 18.5–22.9 kg/m^2^), 25.7% were overweight (BMI 23.0–24.9 kg/m^2^), 29.9% were obese (BMI 25.0–29.9 kg/m^2^), and 3.4% were severely obese (BMI ≥ 30.0 kg/m^2^). The mean WC was 80.8 ± 8.9 cm. For a median follow-up time was 4.39 years (3.31–5.17 years), a total of 50,464 subjects (0.4%) had newly diagnosed thyroid cancer.

**Table 1:** Baseline characteristics of subjects.

	Total	Male	Female
*N* (%)	11,323,006 (100)	6,286,343 (56)	5,036,663 (44)
Age (yr)	50.1 ± 13.7	48.6 ± 13.3	52.1 ± 13.9
Height (cm)	163.6 ± 9.3	169.7 ± 6.4	156.0 ± 6.2
Weight (kg)	64.1 ± 11.5	70.0 ± 10.4	56.8 ± 8.3
BMI (kg/cm^2^)	23.8 ± 3.2	24.2 ± 3.0	23.3 ± 3.3
BMI category (kg/cm^2^)			
<18.5	372,964 (3.3)	127,374 (2.0)	245,590 (4.9)
18.5–23	4,277,580 (37.8)	2,036,484 (32.4)	2,241,096 (44.5)
23–25	2,905,362 (25.7)	1,745,214 (27.8)	1,160,148 (23.0)
25–30	3,380,896 (29.9)	2,156,340 (34.3)	1,224,556 (24.3)
≥30	386,204 (3.4)	220,931 (3.5)	165,273 (3.3)
WC (cm)	80.8 ± 8.9	84.0 ± 7.7	76.9 ± 8.8
WC category (male/female) (cm)			
<80/75	3,832,558 (33.9)	1,699,595 (27.0)	2,132,963 (42.4)
−85/80	2,748,903 (24.3)	1,697,214 (27.0)	1,051,689 (20.9)
−90/85	2,381,362 (21.0)	1,480,917 (23.6)	900,445 (17.9)
−95/90	1,403,792 (12.4)	876,655 (14.0)	527,137 (10.5)
−100/95	624,926 (5.5)	359,125 (5.7)	265,801 (5.3)
≥100/95	331,465 (2.9)	172,837 (2.8)	158,628 (3.2)
Smoking			
non-smoker	6,813,661 (60.2)	1,960,324 (31.2)	4,853,337 (96.4)
ex-smoker	1,853,216 (16.4)	1,784,410 (28.4)	68,806 (1.4)
current smoker	2,656,129 (23.5)	2,541,609 (40.4)	114,520 (2.3)
Alcohol intake			
none	6,010,066 (53.1)	2,060,283 (32.8)	3,949,783 (78.4)
mild (1–3 times/month)	4,588,581 (40.5)	3,532,283 (56.2)	1,056,298 (21.0)
moderate (≥1 time/week)	724,359 (6.4)	693,777 (11.0)	30,582 (0.6)
Regular physical activity (yes)	6,043,929 (53.4)	3,775,278 (60.0)	2,268,651 (45.0)
Diabetes mellitus	1,106,203 (9.8)	680,418 (10.8)	425,785 (8.5)
Hypertension	3,244,666 (28.7)	1,842,107 (29.3)	1,402,559 (27.9)
Dyslipidemia	2,407,672 (21.3)	1,190,861 (18.9)	1,216,811 (24.2)
Chronic kidney disease	721,443 (6.4)	347,662 (5.5)	373,781 (7.4)
Place (urban)	5,055,577 (44.7)	2,759,282 (43.9)	2,296,295 (45.6)
Thyroid cancer (newly diagnosed)	50,464 (0.4)	14,765 (0.2)	35,699 (0.7)

Continuous variables are expressed as means ± standard deviations.

Categorical variables are presented as numbers (percentages).

BMI, body mass index; WC, waist circumference.

Regular physical activity was defined as more than one day of moderate-intensity or vigorous intensity in a week using self-reporting questionnaires.

### Incidence of thyroid cancer according to BMI and WC categories

**Figure 1:**
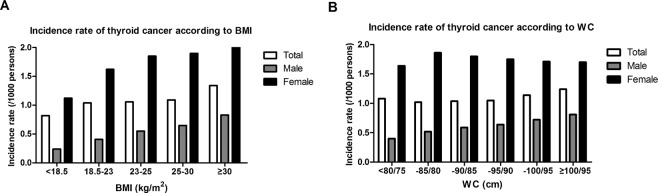
Incidence rates of thyroid cancer according to (**A**) body mass index and (**B**) waist circumference.

We evaluated the incidence of thyroid cancer according to BMI categories (Table 2). After adjusting for age, sex, smoking, alcohol intake, regular physical activity, diabetes, hypertension, and dyslipidemia, the incidence of thyroid cancer significantly increased as BMI increased (*P* for trend < 0.001, Table 2, Fig. 1A). There were dose-response association between BMI and the incidence of thyroid cancer in both males and females.

**Table 2:** Incidence of thyroid cancer according to body mass index and waist circumference categories.

	Incidence rate	Hazard ratio	*P* for trend
**Total**			
BMI category (kg/m^2^)			<0.001
<18.5	0.82	0.64 (0.61–0.68)	
18.5–22.9	1.04	1 (reference)	
23–24.9	1.06	1.26 (1.23–1.29)	
25.0–29.9	1.09	1.38 (1.35–1.41)	
≥30.0	1.34	1.52 (1.45–1.59)	
WC category (Male/Female cm)			<0.001
<80/75	1.08	1 (reference)	
−85/80	1.02	1.27 (1.24–1.30)	
−90/85	1.04	1.36 (1.32–1.39)	
−95/90	1.05	1.40 (1.36–1.45)	
−100/95	1.14	1.46 (1.40–1.52)	
≥100/95	1.24	1.48 (1.40–1.56)	
**Male**			
BMI category (kg/m^2^)			<0.001
10.4–18.5	0.24	0.64 (0.54–0.75)	
18.5–22.9	0.41	1 (reference)	
23–24.9	0.55	1.30 (1.24–1.36)	
25.0–29.9	0.65	1.52 (1.46–1.59)	
30.0–64.1	0.83	1.89 (1.74–2.05)	
WC category (cm)			<0.001
52–80	0.40	1 (reference)	
80–85	0.52	1.31 (1.25–1.38)	
85–90	0.59	1.52 (1.44–1.59)	
90–95	0.64	1.68 (1.58–1.78)	
95–100	0.72	1.89 (1.76–2.03)	
100–121	0.81	2.10 (1.92–2.30)	
**Female**			
BMI category (kg/m^2^)			<0.001
10.6–18.5	1.12	0.64 (0.60–0.67)	
18.5–22.9	1.62	1 (reference)	
23–24.9	1.85	1.26 (1.22–1.29)	
25.0–29.9	1.90	1.33 (1.30–1.37)	
30.0–55.3	2.01	1.39 (1.31–1.47)	
WC category (cm)			<0.001
52–75	1.64	1 (reference)	
75–80	1.86	1.22 (1.19–1.26)	
80–85	1.80	1.26 (1.22–1.29)	
85–90	1.75	1.25 (1.21–1.30)	
90–95	1.71	1.26 (1.20–1.32)	
95–121	1.70	1.24 (1.16–1.32)	

Hazard ratio (95% Confidence interval) was adjusted for age, sex, smoking, alcohol intake, regular physical activity, diabetes, hypertension, and dyslipidemia.

BMI, body mass index; WC, waist circumference.

Incidence of thyroid cancer was analyzed according to WC categories in both males and females (Table 2). In multivariable analysis, the incidence of thyroid cancer significantly increased in subjects as WC increased in both males and females (*P* for trend < 0.001, Table 2, Fig. 1B). The HRs for males for each WC category were as follows: WC < 80 cm (reference), 80–85 cm (HR 1.31 [1.25–1.38]), 85–90 cm (HR 1.52 [1.44–1.59]), 90–95 cm (HR 1.68 [1.58–1.78]), 95–100 cm (HR 1.89 [1.76–2.03]), and ≥100 cm (HR 2.10 [1.92–2.30]). The HRs for females for each WC category were as follows: WC < 75 cm (reference), 75–80 cm (HR 1.22 [1.19–1.26]), 80–85 cm (HR 1.26 [1.22–1.29]), 85–90 cm (HR 1.25 [1.21–1.30]), 90–95 cm (HR 1.26 [1.20–1.32]), and ≥95 cm (HR 1.24 [1.16–1.32]).

When we performed an additional analysis after excluding underweight participants, the results showed similar trends (Supplementary Table 1).

### Risk of thyroid cancer according to weight change

**Figure 2:**
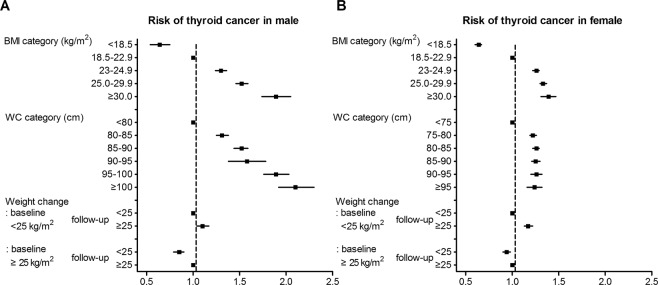
Risk of thyroid cancer in (**A**) males and (**B**) females.

We evaluated the association between weight change and the incidence of thyroid cancer (Table 3 and Fig. 2). When lean subjects without weight change for four years were defined as the reference group, the incidence of thyroid cancer increased significantly in subjects who were lean and became obese (HR 1.10 [1.04–1.17], *P* < 0.001) in males. When obese subjects without weight change for four years were defined as the reference group, the incidence of thyroid cancer decreased significantly in subjects who were obese and became lean in males (HR 0.85 [0.79–0.90], *P* < 0.001).

**Table 3:** Risk of thyroid cancer according to weight change.

BMI (kg/m^2^)		Total		Male		Female	
4 years prior to the baseline	baseline	HR (95% CI) (proportion)	*P*	HR (95% CI) (proportion)	*P*	HR (95% CI) (proportion)	*P*
<25	<25	1 (reference) (61.0%)	<0.001	1 (reference) (56.2%)	<0.001	1 (reference) (66.9%)	<0.001
	≥25	1.15 (1.11–1.19) (6.9%)		1.10 (1.04–1.17) (7.8%)		1.17 (1.13–1.22) (5.8%)	
≥25	<25	0.89 (0.86–0.93) (5.8%)	<0.001	0.85 (0.79–0.90) (6.0%)	<0.001	0.94 (0.90–0.98) (5.5%)	<0.001
	≥25	1 (reference) (26.4%)		1 (reference) (30.0%)		1 (reference) (21.8%)	

Hazard ratio (95% Confidence interval) was adjusted for age, sex, smoking, alcohol intake, regular physical activity, diabetes, hypertension, and dyslipidemia.

BMI, body mass index.

In females, the incidence of thyroid cancer increased significantly in subjects who were lean and became obese (HR 1.17 [1.13–1.22], *P* < 0.001). In subjects who were obese and became lean, the incidence of thyroid cancer decreased significantly (HR 0.94 [0.90–0.98], *P* < 0.001).

When we performed an additional analysis after excluding underweight participants, the results showed similar trends (Supplementary Table 2).

## Discussion

In this study, we aimed to evaluate the association between obesity and the incidence of thyroid cancer using a large population-based cohort from the Korean National Health Screening database after excluding participants with hypothyroidism or hyperthyroidism at initial enrollment. Higher BMI and larger WC were significantly associated with an increased risk of thyroid cancers in both males and females. Higher BMI and larger WC had stronger associations with the incidence of thyroid cancers in males than in females. Weight gain in lean subjects was associated with increased risk of thyroid cancers, and weight reduction in obese subjects was associated with decreased risk of thyroid cancers.

Recently, several studies reported that obesity was positively associated with an increase in the risk of thyroid cancer^3,14–21^. A prospective cohort study showed that subjects with BMIs 25.0–29.9 and ≥30 kg/m^2^ compared to subjects with BMIs between 18.5–24.9 kg/m^2^ had an increased risk of thyroid cancer (relative risk 1.27 and 1.39, respectively)^20^. A large pooled analysis of 22 prospective studies had suggested that a high BMI (per 5 kg/m^2^) was associated with an increased risk of thyroid cancer (HR 1.06 [95% CI 1.02–1.10])^21^.

Although BMI is a main surrogate marker for obesity, it cannot distinguish lean body mass from body fat mass or reflect abdominal adiposity^21–24^. WC has stronger correlations with metabolically active visceral adipose tissue than BMI and could reflect risks for cardiovascular disease, all-cause mortality, and cancer^21–25^. There have been few studies on the association of WC and the risk of thyroid cancer, and the results have been conflicting ^18,21,26,27^. The pooled analysis suggested that larger WC (per 5 cm) was associated with an increased risk of thyroid cancer (HR 1.03 [95% CI 1.01–1.05])^21^. A previous prospective study demonstrated that subjects with large WC (>102 cm in males and >88 cm in females) had significantly higher incidences of thyroid cancer in both males and females^26^. However, another prospective study reported that WC had no significant association with the risk of thyroid cancer^27^. In the current study, the incidence of thyroid cancer significantly increased in subjects with larger WC in both males and females after multivariable analysis (*P* for trend < 0.001, Table 2). For males in particular, the risk of thyroid cancer was 2.10 times higher in subjects with WC ≥ 100 cm than in subjects with WC < 80 cm. In females with WC ≥ 95 cm, HR was 1.24 compared to the reference but lower than that of subjects with WC 90–95 cm. It might be associated with the low prevalence of females with WC ≥ 95 cm (Table 1).

In this study, weight gain in lean subjects was associated with an increase in the risk of thyroid cancer for both males (HR 1.10 [1.04–1.17], *P* < 0.001) and females (HR 1.17 [1.13–1.22], *P* < 0.001), which is consistent with previous studies^26,29^. A previous case-control study showed that subjects with a total weight gain ≥ 10 kg had a higher incidence of thyroid cancer (men, odds ratio [OR] 5.39, [95% CI 3.88–7.49]; women, OR 3.36 [95% CI 2.87–3.93]) than subjects with a stable weight (loss or gain < 5 kg)^29^. A marked increase in BMI starting at age 35 years (annual average increase in BMI ≥ 0.3 kg/m^2^/year) was related to an increased risk of thyroid cancer^29^. However, a weight reduction (annual average decrease in BMI ≥ 0.1 kg/m^2^/year) was not associated with a decreased risk of thyroid cancer in both males (OR 0.58 [95% CI 0.31–1.04]) and females (OR 1.02 [95% CI 0.79–1.32])^29^. Another prospective study had reported a positive association between weight gain for males age 18–35 years and risk of thyroid cancer (gained ≥ 10.0 kg vs. lost/gained < 5 kg, HR 1.49 [95% CI 0.93–2.39], *P* for trend = 0.03)^26^. However, weight gain between the ages of 18 to the current age, 35 to 50, and 50 to current age had no significant association in males and had even less pronounced association in females^26^. Weight reduction between the ages 18 to the current age, 18 to 35, 35 to 50, and 50 to the current age were not significantly associated with a decreased risk of thyroid cancer in both males and females in that study^26^. In the present study, when obese subjects without weight change for 4 years were defined as the reference group, the incidence of thyroid cancer decreased significantly in subjects who were obese and became lean in both males (HR 0.85 [0.79–0.90], *P* < 0.001) and females (HR 0.94 [0.90–0.98], *P* = 0.004). These findings suggest that weight reduction in obese subjects could decrease their risk of thyroid cancer.

Several potential mechanisms such as inflammation, oxidative stress, altered immune response, increased thyroid stimulating hormone (TSH) levels, hyperinsulinemia, adipokines, and increased aromatase activity have been suggested to explain the relationship between obesity and thyroid carcinogenesis^5,26,29,39–42^. Chronic low-grade inflammation has been associated with increased formation of reactive oxygen species, increased cell cycle rate, and decreased tumor suppressor function^5,40^. Increased serum TSH levels could stimulate proliferation and growth of thyroid cells, increased mutation, and the development of thyroid cancer^26,39,40^. Hyperinsulinemia and increased insulin-like growth factor 1 (IGF-1), which is a result of insulin resistance, is another hypothesis for thyroid carcinogenesis^5,29,39,40^. By binding to the insulin receptor, insulin activates downstream AKT/mTOR/PI3K and ERK/RAS/MAPK pathways which are involved in cancer proliferation and survival^40^. Adipokines including adiponectin, leptin, and resistin could affect thyroid carcinogenesis^39,40^. Because the underlying mechanism is not completely understood, further studies on the association between obesity and thyroid cancer are needed.

Consistent with previous studies, males in our study showed a stronger association between obesity and risk of thyroid cancer than females^21,26,29,30^. The risk of thyroid cancer was 1.89 and 1.39 times higher in severely obese subjects (BMI ≥ 30.0 kg/cm^2^) than in normal-weight subjects (BMI 18.5–22.9 kg/cm^2^) in males and females, respectively. The risk of thyroid cancer was 2.10 times higher in males with WC ≥ 100 cm than in males with WC < 80 cm, and the risk was 1.24 times higher in females with WC ≥ 95 cm than in females with WC < 75 cm. A previous study demonstrated that the risk of thyroid cancer increased with WC, which was categorized into quartiles (*P* for trend = 0.007), however the trend was not statistically significant in females (*P* for trend = 0.13)^26^. On the other hand, other studies have reported a positive correlation between obesity and risk of thyroid cancer only in females^15,18^. Using data from subjects who underwent a routine health checkup, the prevalence of thyroid cancer was associated with a high BMI only in females (per 5 kg/m2 increase, OR 1.63 [95% CI 1.24–2.10], *P* < 0.001)^15^. The reasons for these sex differences are not fully understood. Hormone dimorphism, differences in body fat distribution, and metabolic consequences have been proposed to explain sex- dependent associations between obesity and risk of thyroid cancer^26,29,39^.

This study has several limitations. Because of its retrospective population-based design, this study could have a possibility several biases including coding bias, selection bias, and effects of confounding factors. A possibility that overweight or obese adults are more likely to undergo diagnostic examination or cancer screening cannot be excluded. It could be a problem in establishing a causal relationship. Because the NHIS database depends on a diagnostic code for thyroid cancer submitted on the physician’s claim, we could not evaluate the types of thyroid cancer, clinic-pathological features such as tumor size or nodal metastasis. Papillary and follicular thyroid cancer account for most of the thyroid cancer in Korea, however, the impact of medullary or anaplastic thyroid cancer might be small in this study^43,44^. Also, we could not obtain the information about how the thyroid cancers were detected. Data from thyroid function tests were not available in this study. We assessed weight changes as a relatively short period of 4 years, and could not know whether the weight changes were intentional or unintentional. Information on diet or energy intake was not available, so we could not adjust these variables. The results of this study may not be generally applicable to other populations, because of the widespread use of thyroid ultrasonography in Korea^11^. Nevertheless, this study evaluated the effect of WC and weight change the risk of thyroid cancer using a nationwide population-based cohort from on the national health insurance database. We adjusted for potential confounding factors, such as smoking status, alcohol intake, physical activity, diabetes, hypertension, and dyslipidemia.

In this nationwide, population-based cohort study, higher BMI and larger WC were significantly associated with the increased risk of thyroid cancers in both males and females after adjustment for smoking status, alcohol intake, physical activity, diabetes, hypertension, and dyslipidemia. Weight gain in lean subjects was associated with an increased risk of thyroid cancers, and weight reduction in obese subjects was associated with decreased risk of thyroid cancer. Further studies are needed to understand the underlying mechanisms about the association between obesity and thyroid cancer.

## Supplementary information

Dataset 1
