# Supplementary material for: Weight change is significantly associated with risk of thyroid cancer: A nationwide population-based cohort study
Source: Sci Rep. 2019 Feb 7;9:1546. doi: 10.1038/s41598-018-38203-0 (PMC6367378; doi:10.1038/s41598-018-38203-0)
Supplement: Supplementary file 1 — Dataset 1 [file 41598_2018_38203_MOESM1_ESM.docx]

**Supplementary Table 1**. **Incidence of thyroid cancer according to body mass index and waist circumference categories after excluding underweight subjects**

|  | **Incidence rate** | **Hazard ratio** | ***P* for trend** |
| --- | --- | --- | --- |
| Total |  |  |  |
| BMI category (kg/m^2^) |  |  | <0.001 |
| 18.5-22.9 | 1.04 | 1 (reference) |  |
| 23-24.9 | 1.06 | 1.26 (1.23-1.29) |  |
| 25.0-29.9 | 1.09 | 1.38 (1.35-1.41) |  |
| 30.0-64.1 | 1.34 | 1.52 (1.45-1.59) |  |
| WC category (Male/ Female cm) |  |  | <0.001 |
| <80/75 | 1.05 | 1 (reference) |  |
| -85/80 | 1.02 | 1.23 (1.20-1.26) |  |
| -90/85 | 1.05 | 1.31 (1.28-1.35) |  |
| -95/90 | 1.05 | 1.36 (1.32-1.41) |  |
| -100/95 | 1.14 | 1.42 (1.36-1.47) |  |
| ≥100/95 | 1.24 | 1.43 (1.36-1.51) |  |
| Male |  |  |  |
| BMI category (kg/m^2^) |  |  | <0.001 |
| 18.5-22.9 | 0.41 | 1 (reference) |  |
| 23-24.9 | 0.55 | 1.30 (1.24-1.36) |  |
| 25.0-29.9 | 0.65 | 1.52 (1.46-1.59) |  |
| 30.0-64.1 | 0.83 | 1.89 (1.74-2.05) |  |
| WC category (cm) |  |  | <0.001 |
| 52-80 | 0.40 | 1 (reference) |  |
| 80-85 | 0.52 | 1.31 (1.25-1.38) |  |
| 85-90 | 0.59 | 1.52 (1.44-1.59) |  |
| 90-95 | 0.64 | 1.68 (1.58-1.77) |  |
| 95-100 | 0.72 | 1.89 (1.76-2.03) |  |
| 100-121 | 0.81 | 2.10 (1.92-2.30) |  |
| Female |  |  |  |
| BMI category (kg/m^2^) |  |  | <0.001 |
| 18.5-22.9 | 1.62 | 1 (reference) |  |
| 23-24.9 | 1.85 | 1.26 (1.22-1.29) |  |
| 25.0-29.9 | 1.90 | 1.33 (1.30-1.37) |  |
| 30.0-55.3 | 2.01 | 1.39 (1.31-1.47) |  |
| WC category (cm) |  |  | <0.001 |
| 52-75 | 1.60 | 1 (reference) |  |
| 75-80 | 1.86 | 1.22 (1.19-1.26) |  |
| 80-85 | 1.80 | 1.26 (1.22-1.29) |  |
| 85-90 | 1.75 | 1.25 (1.22-1.30) |  |
| 90-95 | 1.71 | 1.26 (1.20-1.32) |  |
| 95-121 | 1.70 | 1.24 (1.16-1.32) |  |

Hazard ratio (95% Confidence interval) was adjusted for age, sex, smoking, alcohol intake, regular physical activity, diabetes, hypertension, and dyslipidemia

BMI, body mass index; WC, waist circumference

**Supplementary Table 2.** **Risk of thyroid cancer according to weight change after excluding underweight subject**

| BMI (kg/m^2^) | | Total | | Male | | Female | |
| --- | --- | --- | --- | --- | --- | --- | --- |
| 4 years  prior to  the baseline | baseline | HR (95% CI)  (proportion) | *P* | HR (95% CI)  (proportion) | *P* | HR (95% CI)  (proportion) | *P* |
| <25 | <25 | 1 (reference)  (59.7%) | <0.001 | 1 (reference)  (55.3%) | <0.001 | 1 (reference)  (65.2%) | <0.001 |
|  | ≥25 | 1.15 (1.11-1.19)  (7.1%) |  | 1.10 (1.03-1.17)  (8.0%) |  | 1.19 (1.14-1.24) (6.1%) |  |
| ≥25 | <25 | 0.89 (0.86-0.93)  (6.0%) | <0.001 | 0.84 (0.78-0.90)  (6.1%) | <0.001 | 0.93 (0.88-0.97)  (5.8%) | <0.001 |
|  | ≥25 | 1 (reference) (27.2%) |  | 1 (reference)  (30.6%) |  | 1 (reference) (22.9%) |  |

Hazard ratio (95% Confidence interval) was adjusted for age, sex, smoking, alcohol intake, regular physical activity, diabetes, hypertension, and dyslipidemia

BMI, body mass index
